# Chronic Stanford Type A Aortic Dissection Complicated by Secondary Tracheomalacia

**DOI:** 10.1016/j.jaccas.2025.106744

**Published:** 2026-01-28

**Authors:** Alexis Edmonson, Abhinav Saxena, Niti Dalal, Jamil Borgi, Aabha Divya

**Affiliations:** a Division of Cardiothoracic Surgery, Department of Surgery, Tulane University School of Medicine, New Orleans, Louisiana, USA; b East Jefferson General Hospital, Metairie, Louisiana, USA; c Department of Epidemiology, Tulane School of Public Health and Tropical Medicine, New Orleans, Louisiana, USA

**Keywords:** aorta, computed tomography, dissection, risk factor

## Abstract

**Background:**

Tracheomalacia is an uncommon airway disorder, and vascular compression is a rare underlying cause. Reports of tracheomalacia associated with chronic aortic dissection are exceedingly limited.

**Case Summary:**

A 46-year-old woman with hypertension, stroke with residual left-sided weakness, HIV, and chronic kidney disease presented with chronic aortic dissection of an 8.8-cm ascending aortic aneurysm. She underwent elective ascending aortic hemiarch replacement. Her postoperative course was complicated by delayed extubation and recurrent hypoxic respiratory failure. Bronchoscopy revealed previously undiagnosed severe tracheomalacia, necessitating tracheostomy and prolonged ventilatory support. She gradually improved and was discharged to rehabilitation.

**Discussion:**

This case highlights that the early recognition of airway collapse in patients with large thoracic aneurysms is crucial to prevent extubation failure and optimize postoperative care.

**Take-Home Message:**

Early postoperative extubation failure in patients with large thoracic aneurysms should prompt evaluation for airway collapse. Prompt bronchoscopic assessment is important for planning management.

## Visual Summary

**Figure:**
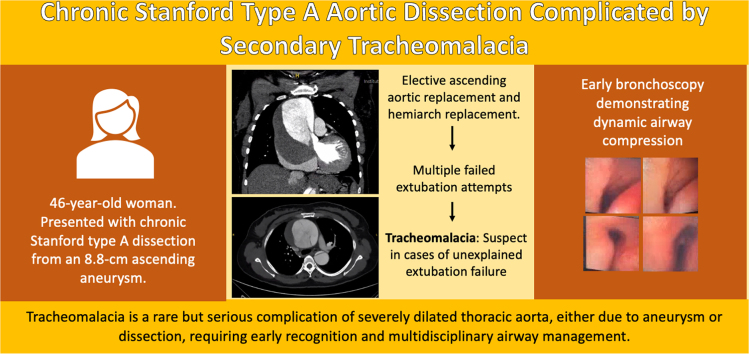

Tracheomalacia is a condition in which the trachea collapses upon itself during expiration. There are several causes of tracheomalacia, including tracheal wall malformation or chronic external compression of the trachea acquired over time.^1^ It occurs in both a dynamic and static state. In the dynamic form, the tracheal lumen is almost completely obstructed throughout the normal cycle of breathing. Congenital forms of tracheomalacia are caused by weakening of the wall of the trachea, which leads to a change in the size and shape of the lumen.^2^ Although congenital forms are well recognized, acquired tracheomalacia in adults most often results from prolonged intubation, chronic inflammation, or extrinsic vascular compression. The latter is an exceedingly rare etiology. External compression increases tracheal compliance and decreases tracheal wall integrity. Diagnosis of this condition is based mostly on clinical symptoms of airway collapsibility combined with pulmonary function testing, imaging, and direct bronchoscopy. Given the ability to directly visualize the airway during inspiration and expiration, bronchoscopy is considered the gold standard for diagnosing tracheomalacia.

**Take-Home Messages**

• Tracheomalacia is a rare complication of thoracic aortic aneurysm or intramural hematoma and should be suspected in patients with unexplained extubation failure.
• Early bronchoscopy and multidisciplinary collaboration are essential for diagnosis and management, and strategies range from noninvasive ventilatory support and tracheostomy to possible extracorporeal membrane oxygenator.

Thoracic aortic aneurysms may be either symptomatic, manifesting with chest pain, dyspnea, or compressive features, or asymptomatic, discovered incidentally on imaging. However, when complicated by acute Stanford type A dissection, untreated mortality is strikingly high, estimated to increase by 1%–2% per hour after symptom onset, reaching nearly 50% at 48 hours without surgical repair.^3^^,^^4^ While many patients succumb early, a subset survives the acute event and progresses to chronic dissection, typically defined as >14 days after onset.^5^ In this phase, remodeling of the thrombosed false lumen may exert compressive effects on adjacent mediastinal structures, including the airway.^6^ We present a case of a patient diagnosed with chronic aortic dissection in a massively dilated aorta, causing secondary tracheomalacia.

## Case Summary

**Figure 1:**
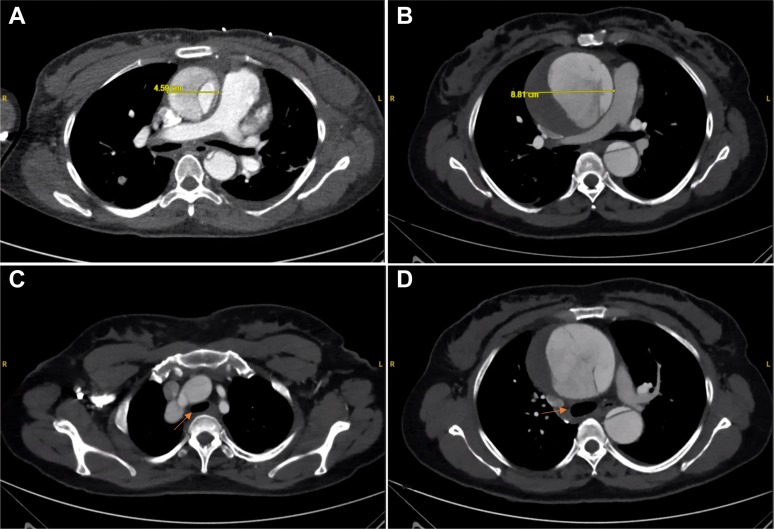
Contrast-Enhanced Computed Tomography With Evolution of Dissection and Tracheomalacia (A) Contrast-enhanced computed tomography angiography (CTA) of the chest in 2019 demonstrating a Stanford type A dissection with an ascending aortic aneurysm measuring 4.5 cm. (B) Follow-up CTA in 2025 showing interval enlargement of the ascending aortic aneurysm to 8.8 cm, with a persistent chronic dissection flap and a large hematoma present in the false lumen. (C) Axial CTA slice highlighting extrinsic tracheal compression from the aneurysmal aorta (arrow), consistent with airway narrowing. (D) Axial CTA slice at the level of the ascending aorta, showing severe airway compromise with compression of the distal trachea and proximal mainstem bronchi (arrow).

**Figure 2:**
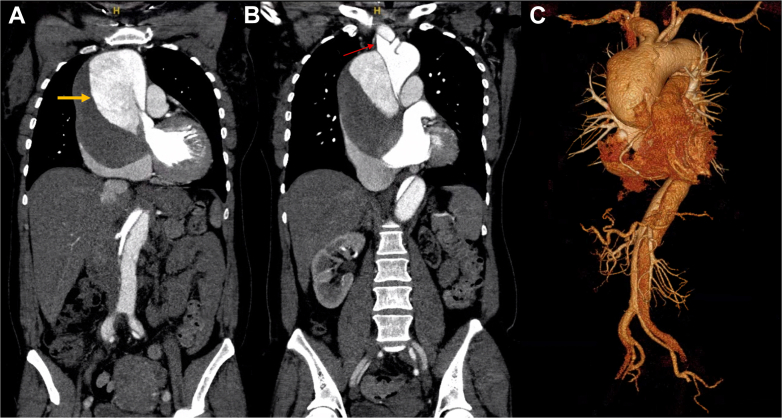
Preoperative Computed Tomography Angiography Demonstrating Chronic Stanford Type A Aortic Dissection With Thrombosed False Lumen (A) Coronal computed tomography angiography (CTA) demonstrating a chronic type A dissection with aneurysmal dilation of the ascending aorta and thrombosed false lumen (arrow). (B) Coronal CTA slice showing arch vessels arising from the true lumen (arrow). (C) Three-dimensional reconstruction of the thoracic and abdominal aorta demonstrating the extent of the dissection flap and aneurysmal dilation of the ascending aorta with distal extension into the abdominal aorta.

A 46-year-old female presented with vague chest pain to the outpatient clinic for chronic Stanford type A aortic dissection (TAAD) with a massively dilated aorta. Her past medical history was significant for hypertension, cardiac arrest in 2022 complicated by stroke with left-sided upper extremity weakness, chronic anemia secondary to hemodialysis for end-stage renal disease, now with chronic kidney disease stage 3, history of amphetamines and cocaine use, and controlled HIV infection on antiretroviral treatment. She had previously undergone computed tomography with angiography in 2019 in an outside hospital, which demonstrated an acute TAAD with an aneurysmal ascending aorta (4.6 × 4.8 cm), an arch diameter of 3.7 cm, and dissection extending into the innominate artery, with evidence of hypoperfusion of the left kidney due to malperfusion from the false lumen (Figure 1A). At that time, she was managed nonoperatively with medical therapy. Subsequently, in 2022, she suffered a cardiac arrest, complicated by cerebrovascular accident, and was not offered surgery. Her repeat computed tomography with angiography in 2022 revealed further dilation of the ascending aorta to 7.8 × 6.9 cm with a large intramural thrombus in the false lumen (Figure 1B). On her referral to the clinic in July 2025, her interval imaging showed progression of the known dissection with further enlargement of the ascending aorta to 8.7 × 8.8 cm (Figures 2C and 2D).

**Figure 3:**
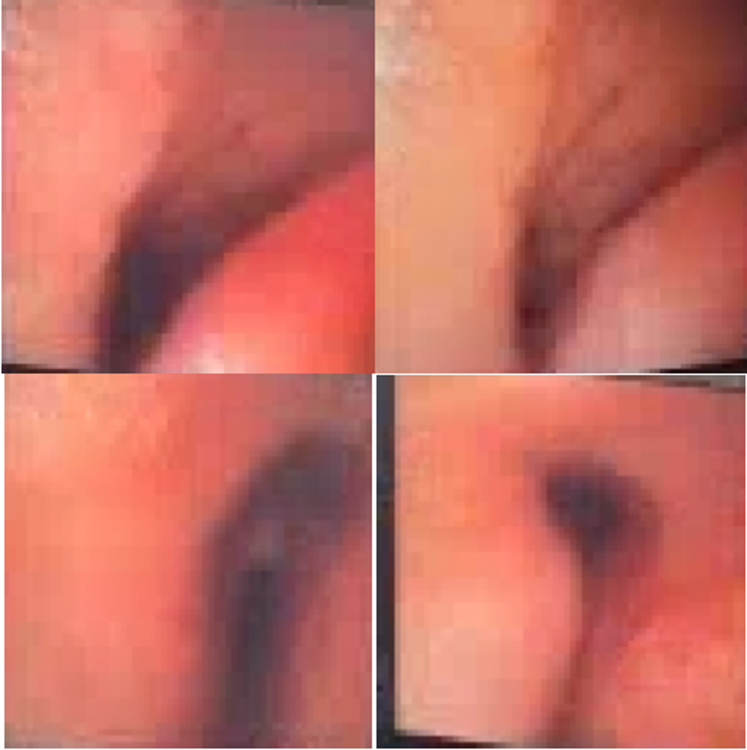
Bronchoscopic Views Demonstrating Dynamic Airway Collapse During Expiration Consistent With Tracheomalacia

She successfully underwent an elective ascending aortic and hemiarch replacement in July 2025. She had a Society of Thoracic Surgeons–predicted morbidity and mortality of 32.6%. Her immediate postoperative course was uncomplicated; however, she experienced failure of extubation due to acute hypoxemic respiratory failure. After failed attempts at extubation, bronchoscopy was performed on postoperative day 5 to evaluate for the underlying cause, which revealed moderate to severe tracheomalacia (Figure 3). The patient did not have a prior known diagnosis of this condition. Subsequent evaluation by interventional pulmonology revealed extensive dynamic airway collapse. Despite further attempts to extubate, the patient was unable to tolerate due to respiratory distress. The patient underwent tracheotomy and percutaneous endoscopic gastrostomy tube placement on postoperative day 14. Tracheal stenting was considered but deferred, given her ventilatory dependence and limited expected benefit. Over the following weeks, her respiratory status improved, allowing transfer to inpatient rehabilitation on postoperative day 46, with planned discharge to home on postoperative day 58.

## Discussion

Tracheomalacia is characterized by excessive collapsibility of the trachea due to loss of structural integrity of the cartilaginous wall. Acquired tracheomalacia most often results from prolonged intubation or chronic inflammation, while vascular compression is an exceedingly rare cause.^1^ In the context of thoracic aortic disease, most reports involve arch or thoracoabdominal aneurysms, where the trachea and bronchi are more directly compressed.^7^, ^8^, ^9^ Tracheomalacia caused by chronic TAAD is exceptionally uncommon (Figure 2).

Given the extensive growth of the patient's aortic aneurysm over time, progressive compression to the trachea likely contributed to her respiratory complications (Figures 1C and 1D). The pathophysiology in such patients is believed to involve chronic extrinsic compression of the airway by the enlarging aneurysm, false lumen, or intramural hematoma. Over time, this external pressure weakens tracheal cartilage, resulting in dynamic collapse that may only become clinically apparent in the perioperative setting. In our patient, this did not become evident until extubation was delayed postoperatively, prompting bronchoscopy evaluation, and was recognized only after repeated extubation failures, consistent with other reports describing delayed airway compromise.^9^

Despite the clinical relevance, tracheomalacia in the setting of ascending aortic aneurysm remains underdiagnosed. In our patient's case, her prolonged extubation course was initially attributed to sedation in the setting of her chronic kidney disease; however, recognition of this complication is critical, as unexplained extubation failure in patients with large aortic aneurysms should raise suspicion for airway collapse. Flexible bronchoscopy remains the diagnostic gold standard, allowing dynamic visualization of the trachea throughout the respiratory cycle. In this case, bronchoscopy on postoperative day 5 revealed moderate to severe tracheomalacia, prompting multidisciplinary management. In patients with known or suspected tracheomalacia, preoperatively, the anesthesia team should be made aware of the potential for airway compromise intraoperatively or postoperatively. Antoine et al. describe the importance of preparedness and forethought from the American Society of Anesthesiologists' Difficult Airway Algorithm^10^ that apply directly to patients with unexpected postoperative airway compromise, particularly delayed extubation.

Treatment of aneurysm-associated tracheomalacia is challenging. Options range from supportive measures, such as extubation to continuous positive airway pressure to ensure airway patency following extubation, to invasive approaches including tracheostomy or tracheal stenting. Although the literature is limited on simultaneous management of ascending aortic aneurysm and tracheomalacia, there have been case reports that describe similar instances of vascular compression leading to secondary tracheomalacia. Komarov et al. described tracheobronchial compression from an aortic arch aneurysm, which resolved with urgent aortic arch replacement.^7^ Ishimine et al. reported a giant thoracoabdominal dissecting aneurysm causing significant airway narrowing. They reported improvement following staged thoracoabdominal aneurysm repair without tracheal intervention.^8^ These cases highlight that tracheomalacia and airway compromise have been most commonly described in the context of arch or thoracoabdominal aneurysms, where the airway is anatomically more vulnerable to external compression.

In patients with profound airway collapse intraoperatively or perioperatively, extracorporeal membrane oxygenation (ECMO) has been described as a bridge to definitive surgical repair. Carlson et al. present a case of a patient with acute TAAD who underwent ascending aorta replacement and subsequently developed aortic arch aneurysm extending into the descending aorta necessitating total arch replacement. During the second operation, progressive hypoxemia and ventilatory failure necessitated initiation of venoarterial ECMO, and bedside bronchoscopy revealed complete airway collapse consistent with acquired tracheomalacia from chronic aneurysmal compression. The patient subsequently underwent tracheal stenting on postoperative day 8 but required reintubation the following day, ultimately necessitating tracheostomy before discharge to a long-term care facility on postoperative day 30.^11^ These cases describe the spectrum of management approaches, from surgical decompression to advanced airway support, based on the severity of airway involvement. Our patient was not placed on ECMO and did not undergo tracheal stenting; however, the trajectory toward tracheostomy underscores a common endpoint in cases of severe aneurysm-associated tracheomalacia.

**Visual Summary:**
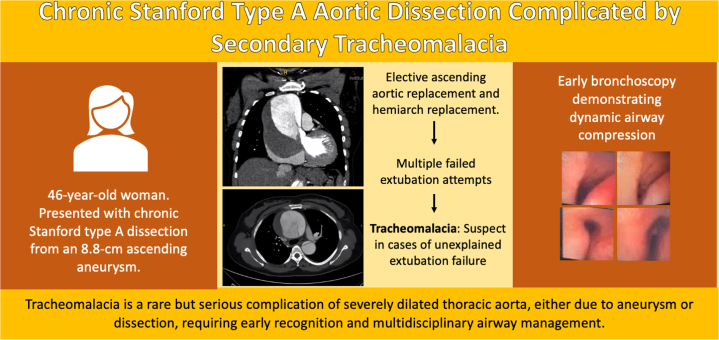
Chronic Stanford Type A Dissection Complicated by Tracheomalacia, Highlighting Progression From Aneurysm to Airway Collapse and the Role of Early Bronchoscopy With Multidisciplinary Management

## Conclusion

Tracheomalacia is an uncommon but clinically significant complication of ascending aortic disease. In patients with unexplained extubation failure following aneurysm repair, airway collapse should be suspected, and early bronchoscopy pursued for diagnosis. Recognition of this rare association can improve perioperative planning and optimize outcomes in patients with thoracic aortic disease.

## Funding Support and Author Disclosures

The authors have reported that they have no relationships relevant to the contents of this paper to disclose.
